# Freshwater Mussels as Sentinels for Safe Drinking Water Supply in Europe

**DOI:** 10.1021/acsestwater.3c00012

**Published:** 2023-11-08

**Authors:** Noé Ferreira-Rodríguez, Sebastian Beggel, Juergen P. Geist, Vanessa Modesto, Martin Österling, Nicoletta Riccardi, Ronaldo Sousa, Maria Urbańska

**Affiliations:** † Universidade de Vigo, Departamento de Ecoloxía e Bioloxía Animal, Facultade de Bioloxía, Campus As Lagoas − Marcosende, Vigo 36310, Spain; ‡ Faculty of Natural and Agricultural Sciences, Ovidius University Constanţa, 900470 Constanţa, Romania; § Aquatic Systems Biology Unit, TUM School of Life Sciences, Technical University of Munich, 85354 Freising, Germany; ⊥ CNR - The National Research Council, IRSA - Water Research Institute, Largo Tonolli 50, 28922 Verbania, Italy; ∥ Institution of Environmental and Life Sciences, Karlstad University, 651 88 Karlstad, Sweden; # CBMA - Centre of Molecular and Environmental Biology, Department of Biology, University of Minho, Campus Gualtar, 4710-057 Braga, Portugal; ∇ Department of Zoology, Poznań University of Life Sciences, Wojska Polskiego 28, 60-637 Poznań, Poland

## Abstract

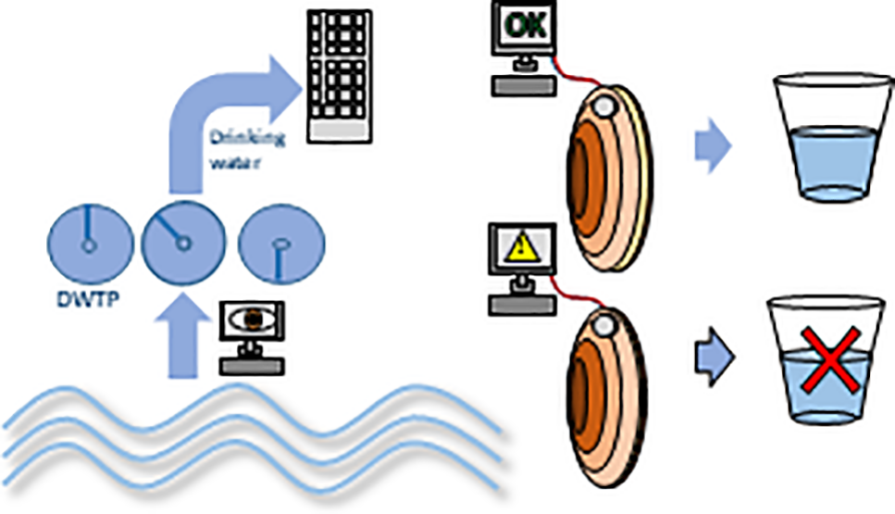

In the context of the European Union (EU) Drinking Water Directive, freshwater mussels (Order Unionoida: Bivalvia) can help us face the challenges of safe drinking water provisions for all citizens in the EU. Specifically, the implementation of high frequency noninvasive (HFNI) valvometers allows the early detection of eventual pollution events in drinking water treatment plants. Currently real-time behavioral analysis is deployed in a number of EU countries, and we foresee a bright future as new technological advances are developed concerning HFNI valvometers.

## Introduction

In recent years, the planet has been undergoing massive alterations caused by anthropogenic activities, resulting in biodiversity loss and increasing global temperatures, among other changes.^1^ Freshwater ecosystems are considered hotspots of endangerment due to the convergence between their relatively high biodiversity—compared to marine and terrestrial counterparts—and many forms of anthropogenic pressure, with a direct impact on biodiversity, ecosystem services, and human well-being.^2,3^

With more than 350,000 chemicals and mixtures of chemicals registered for production and use worldwide (i.e., chemicals with CAS Registry Numbers),^4^ water pollution is one of the world’s most serious environmental problems we need to face^5^ and is directly connected with human health with respect to safe drinking water provision. Given the variety of chemicals, their mixtures, and possible interactions, it is costly and practically impossible to perform chemical analyses of each of them at a high frequency. Moreover, increasing ambient temperatures and water abstraction for human activities not only lead to water scarcity, but also cause the concentration of contaminants, which will worsen the future scenario.^6^ Under this context, for assessing the toxicological effects of environmental contaminants, lethality (e.g., median lethal concentration, LC50), developmental (e.g., growth rate, morphological abnormalities), and reproductive (e.g., fecundity, hatching) endpoints have been traditionally used in biological early warning systems (BEWSs).^7^ More recently, however, the behavioral response (e.g., swim speed, distance moved, activity levels) of different organisms—including waterfleas, mussels, and fishes—has been widely implemented, especially in Europe and Asia.^8^ In addition, the technological development of data acquisition systems allowed incorporation of automated monitoring and machine learning in unsupervised BEWSs.^9^

Here, in order to facilitate the implementation of the principle of “safe drinking water for all in Europe”,^10^ we summarize the current state of the art of using freshwater mussels (Order Unionoida: Bivalvia) as an integrative tool for water quality monitoring in drinking water treatment plants (DWTPs).

## High Frequency Noninvasive (HFNI) Valvometry with Focus on European Freshwater Mussels

**Figure 1:**
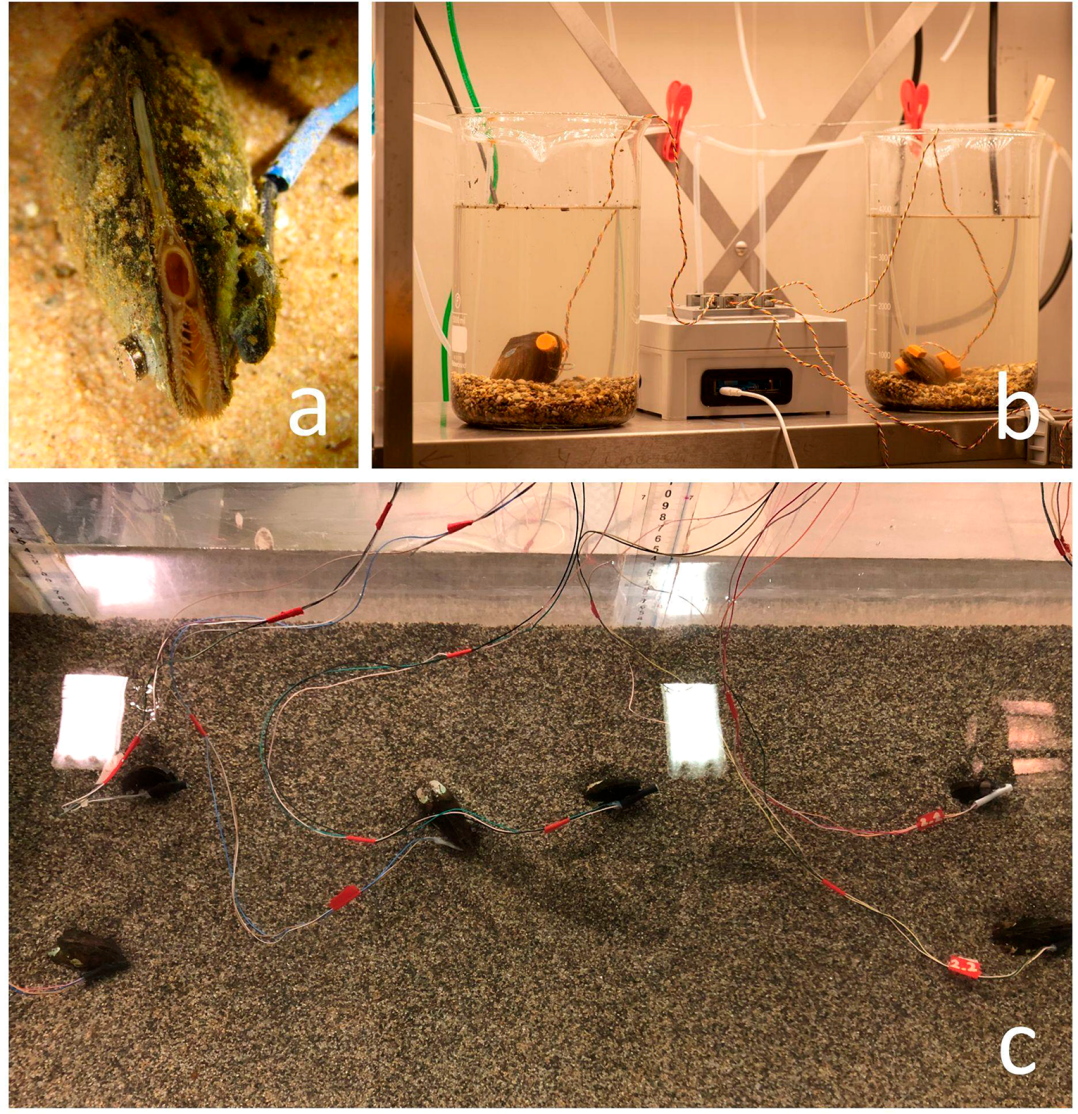
(a) *Unio elongatulus* equipped with a high frequency noninvasive (HFNI) valvometer. (b) Mussel (*Anodonta anatina*) valvometer setup in the laboratory (Aquatic Systems Biology, Technical University of Munich, Germany). (c) Artificial flume used for experimental measurements of mussels’ (*Unio elongatulus*) responses under controlled conditions (University of Trento, Italy).

**Figure 2:**
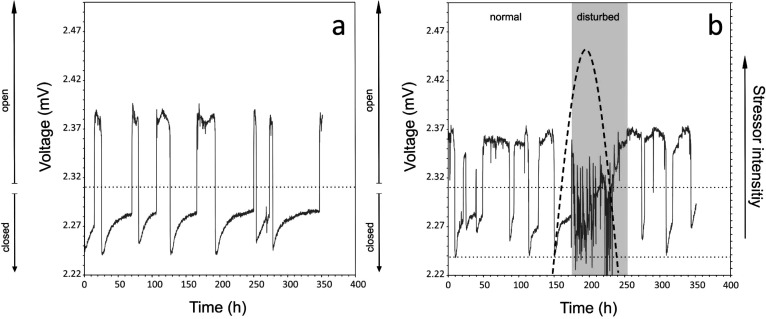
Example time course of mussel behavior measured as Hall voltage (solid line) for (a) normal and (b) stressed conditions. The dashed line exemplarily indicates stressor intensity (i.e., NaCl exposure) causing irregular disturbed valve movements, defined as avoidance behavior. The dotted horizontal line is the mean voltage over the entire experimental period, used to separate open from closed states.

Freshwater mussels are a widely distributed group of relatively long-lived aquatic animals.^11^ Because of their sessile filter feeding behavior, benefits provided by mussels have been related to drinking water production.^12,13^ Freshwater mussels have been also signaled as good sentinels or biomonitors of environmental change,^14^ both concerning long-term and acute responses to environmental stressors.^15,16^ Mussel behavior (e.g., valve gaping) informs about endogenous circadian rhythms, foot extension (foot activity), periods of feeding and respiration, and can be even used to assess exogenous stressful conditions.^17−19^ Valve gaping behavior can be easily monitored by using high frequency noninvasive (HFNI) valvometers (Figure 1), which measure an induced voltage that varies according to the distance between the electromagnetic electrodes. HFNI valvometers are based on the regular gaping of bivalves and the fact that physical (e.g., turbidity)^20,21^ or chemical (e.g., salinity)^16^ stressors disrupt that gaping reference pattern.^22^ These systems employ high frequency electromagnetic induction sensing technology (i.e., a Hall effect sensor) to detect in real-time the duration and magnitude of valve opening occurrence (Figure 2).^23^

Real-time behavioral analysis is used predominantly for sensing water quality entering DWTPs, and it is reportedly deployed in a number of European countries. In DWTPs, mussels are kept in tanks that constantly receive running water from a river via intake stations or tap water to go into the water supply system. Valve gaping behavior is continuously monitored using commercial HFNI valvometers that generate an alarm signal and induce automated sampling if synchronous avoidance behavior is deployed (i.e., synchronous shell closure). In Poland, for example, real-time monitoring by using wild *Unio tumidus* mussels has been implemented nationwide to inform about eventual pollution events in more than 50 DWTPs, which altogether monitor the water consumed by ten million people.^24^ In Italy, the DWTP of Pontelagoscuro (Ferrara province) withdraws raw water directly from the Po River. In 2010, an oil spill into the Lambro River occurred and reached the Po River the following day, compromising the water supply.^25^ For early detecting such events, the DWTP is now equipped with a biological early warning system (BEWS) using individuals of the invasive mussel species *Sinanodonta woodiana* as sentinels, and with lagoon basins, which, if needed, allow the interruption of withdrawals from the Po River and guarantee 3 days of water supply.^26^ Also in Italy, a commercial biological early warning system, the Mosselmonitor, monitors the drinking water supply of Turin.^27^ In Germany, the “Dreissena-Monitor” installed during the 1990s in major rivers for surface water monitoring is, to the best of our knowledge, still in use nationwide.^28^

## Challenges and Opportunities

The current implementation of BEWSs based on HFNI valvometers in DWTPs presents, however, some important challenges. First, HFNI valvometers are commonly attached to wild mussels, which, after three months of use, are returned to the natural environment and replaced with new individuals (e.g., in Poland). However, returning mussels after their use to the natural environment may have unwanted consequences (e.g., pathogens and parasites transmission).^29^ Second, different species may respond disparately to similar environmental changes.^30^ Even within the same species, the behavioral response may vary in relation to season, reproductive status, lentic vs lotic conditions, or other background conditions.^31^ Moreover, different individuals belonging to the same species, in the same season with the same conditions, can respond differently to environmental stressors (i.e., interindividual variation).^32^ As a consequence, to understand behavioral patterns, a reasonable number of individuals may be needed. Third, freshwater mussels are a highly endangered group of aquatic animals. For instance, in Europe, 13 out of 20 unionid mussel species currently considered valid are classified as Threatened or Near Threatened on the IUCN Red List of threatened species.^33,34^ Hence, given their poor conservation status, the use of wild freshwater mussels for water monitoring is highly controversial. An alternative to native mussels may be the use of invasive species that are not underlying any protective measures. However, this situation may also be problematic, increasing the chances of dispersal and possibly even triggering the local human community to protect these harmful species, facilitating their incorporation into the local culture.^35^ Hence, the use of invasive freshwater mussels, such as *S. woodiana* (e.g., in Italy) or *Dreissena polymorpha* (e.g., in Germany) in BEWSs may also have unwanted consequences for conservation management. Fourth, another aspect that needs to be considered is that those mussel species being most sensitive to environmental pollution such as the European freshwater pearl mussel (*Margaritifera margaritifera*) typically only occur in restricted areas in small remnant and protected populations,^36^ which further limit its widespread use as a bioindicator.

The use of captive-breed native mussel species may address most of the flaws mentioned above, providing animals without affecting already threatened wild populations. In this regard, a European network of captive-breeding facilities has been implemented to propagate native freshwater mussels.^37^ This existing network of facilities represents an opportunity to provide animals of known genetic constitution^38^ and similar environmental background to be used in monitoring eventual pollution events in DWTPs. Unfortunately, none of the southern European species (e.g., *Potomida littoralis*, *Microcondylea bonelli*, *Unio elongatus, U. tumidiformis*) are bred. Therefore, captive breeding of more common and widespread species (e.g., *Anodonta anatina*, *Unio pictorum*) may be the most obvious solution and require further research efforts.

Once these challenges are solved, we need to (1) identify potential pollution sources in the target basin, (2) test the behavioral response of the model species to the most common or probable pollutants, including the determination of a minimal sensitivity threshold (i.e., the trace element concentration), and (3) understand how mussels react to mixtures of key pollutants compared to single substances. However, caution must be taken since animals’ behaviors to changes in ambient conditions are highly variable, which could lead to a misinterpretation of observed responses. For instance, mussels’ responses can reflect not only pollutants but also other physicochemical parameters (e.g., discharge variations with sediment transport).^39^ In addition, mussels are sometimes tolerant to persistent pollutants such as DDT, whereas they are very sensitive to others such as dichlorvos, DDVP.^40^ The application of more sophisticated mathematical models therefore needs to be further tested for an array of substances and physicochemical parameters, since it has been previously shown that this could significantly improve the sensitivity of observed stress responses.^17,41^

## HFNI Valvometry in the Future

Although there is a large body of knowledge available for the application of HFNI valvometers as biosensors in BEWSs using marine bivalves,^22^ its use in continental waters is a novel field of research. Technology around HFNI valvometers is, in fact, a work in progress, as new challenges appear in their practical implementation. For instance, open-source platforms such as Arduino or Rasberry Pi are commonly used to control valvometry systems in laboratory monitoring.^42^ Sensors calibration using the Arduino platform is one of the most time-consuming steps; hence, self-calibrating devices and user-friendly free software have been also developed to simplify the use of these systems. One further aspect is the relatively labor intensive manual analysis of the data to identify stressor-related alterations in behavior. Recent advances in automated pattern recognition, such as the use of machine learning and other artificial intelligence (AI) algorithms, could further improve the sensitivity of the systems and reduce the risk of generating false alarms. Wireless monitoring of mussel behavior incorporated in a wider network of different sensors and developing a self-sufficient energy supply for the biosensor technology are also future topics to be explored.^43^ One of the most promising advances in this area is the development of Aqua-Fi systems based on optical—LED or laser light—wireless transmissions as a cost-effective, flexible, and practical methodology for collecting environmental data in real time.^44^ The system as a whole requires further research, but miniaturization and underwater Internet will make possible a proactive management of water resources. With these advances, the applications and capacities of these biosensors will be immeasurable.

## Final Remarks

Altogether, the use of freshwater mussels as biosensors in DWTPs may increase our capacity to safeguard sustainable access to adequate quantities of acceptable quality water, ensuring protection against water-borne pollution as a fundamental part of the so-called water security.^45^ This is especially relevant after the unpreceded catastrophic events (die-offs of fish and freshwater mussel populations) in response to the 2022 drought all across Europe that highlight the demand for action to mitigate the ecological and economic consequences of such extreme events.^46^ Moreover, ensuring access to safe drinking water and sanitation is also one of the 17 Sustainable Development Goals (SDGs) adopted by all United Nations Member States in the 2030 Agenda for Sustainable Development.^47^ Back in Europe, the Parliament formally adopted the revised drinking Water Directive (Directive (EU) 2020/2184)^10^ to ensure access to safe drinking water for all. The Directive entered into force in January 2021, and Member States should transpose it into national legislation in two years, before January 2023. Several Member States failed, however, to notify of national measures fully transposing the Directive, driving the adoption of a package of infringement decisions in March 2023.^48^ In its Article 13, the Directive gives special attention to appropriate monitoring in the catchment areas for abstraction points or in raw water. In this context, we aim to keep the attention on the implementation of technological solutions based on HFNI valvometry systems as a smart, green, and cost-effective alternative for water management. In addition, for its successful implementation, we aim to inspire a call to action in the scientific community, water managers, and policy makers to produce, share, and use research and knowledge to improve water security globally.
